# Mice can recognise water depths and will avoid entering deep water

**DOI:** 10.1515/tnsci-2020-0208

**Published:** 2022-01-31

**Authors:** Hiroshi Ueno, Yu Takahashi, Shunsuke Suemitsu, Shinji Murakami, Naoya Kitamura, Kenta Wani, Yosuke Matsumoto, Motoi Okamoto, Takeshi Ishihara

**Affiliations:** Department of Medical Technology, Kawasaki University of Medical Welfare, Okayama 701-0193, Japan; Department of Psychiatry, Kawasaki Medical School, Kurashiki 701-0192, Japan; Department of Neuropsychiatry, Graduate School of Medicine, Dentistry and Pharmaceutical Sciences, Okayama University, Okayama 700-8558, Japan; Department of Medical Technology, Graduate School of Health Sciences, Okayama University, Okayama 700-8558, Japan

**Keywords:** anxiety-like behaviours, mouse, phobia, water

## Abstract

Rodents are averse to bodies of water, and this aversion has been exploited in experiments designed to study stress in mice. However, a few studies have elucidated the characteristics of murine water aversion. In this study, we investigated how mice behave in and around areas filled with water. Using variants of the open field test that contained pools of water at corners or sides of the field, we recorded the movements of mice throughout the field under various conditions. When the water was 8 mm deep, the mice explored the water pool regardless of whether an object was placed within it, but when the water was 20 mm deep, the mice were less willing to enter it. When the mice were placed on a dry area surrounded by 3 mm-deep water, they explored the water, but when they were surrounded by 8 mm-deep water, they stayed within the dry area. Our results indicate that mice exhibit exploratory behaviours around water, they can recognise water depths and avoid unacceptably deep water, and their willingness to enter water may be reduced by situational anxiety. Our experimental method could be used to investigate water-related anxiety-like behaviours in mice.

## 1 Background

In the wild, mice exhibit a tendency to avoid water as much as possible [1], and when mice are placed in water, they fall and immediately stiffen. Although it is difficult to determine the exact feelings of mice with regard to water, these observations clearly indicate that they do not like water. The aversion of mice to water has been exploited in the design of various behavioural tests, including the forced swim test, the water T-maze test, and the Morris water maze test [2]. Furthermore, this aversion is frequently used to induce chronic stress in mice through repeated forced swimming sessions and the placement of wet rugs in breeding cages [3,4,5].

The irrational fear of water in humans is known as aquaphobia, and aquaphobia is among the common simple phobias. Phobias, which are defined as abnormal psychological and physiological fears for a specific thing [6], are classified as anxiety disorders in the tenth revision of the International Statistical Classification of Diseases and Related Health Problems and are common comorbidities in patients with other anxiety disorders [7,8]. A past investigation [9] reported an aquaphobia prevalence of 1.8%, or approximately 1 in 50 people, in the general Icelandic population, and the symptoms of aquaphobia, which can include headache, suffocation, panic attacks, and decreased water intake [10], can adversely affect productivity, confidence levels, and overall health. However, only 9% of patients with general phobias report having consulted a physician about their conditions [9].

The causes of phobias remain largely undetermined. Researchers have speculated that aquaphobia may arise from a combination of genetics and experiential factors (e.g. swimming ability and instances of needing to be rescued from water) [11]. It is often thought that experiential factors are the most important contributors to aquaphobia in adults, but Poulton et al. found no association between swimming experience during the first 9 years of life and aquaphobia at the age of 18 years [12]. In accordance with Darwin’s non-associative model of fear acquisition, aquaphobia may constitute a type of innate fear that can manifest without any history of distressing experiences. Such innate fears may diminish over time due to repeated safe exposures to fear-inducing stimuli [13], and people can indeed learn to overcome or manage aquaphobia.

The neural mechanisms underlying phobias and innate fears are unknown, and this lack of knowledge has prevented the development of any mechanistic therapies or diagnostic markers for aquaphobia [14]. The advancement of therapeutic strategies for aquaphobia thus depends on the acquisition of experimental evidence identifying the neurochemical and neuroanatomical pathways underlying phobias [15], and given their aversion to water, mice are a tempting model organism for investigations into aquaphobia. However, it is unclear whether the aversion of mice to water truly reflects a fear of water, and certain key parameters of murine water aversion that could clarify the matter remain unexplored. For example, researchers have not fully determined the degree to which mice will modify their behaviours to avoid water, and it remains unknown whether a mouse’s behaviour around a body of water depends on the water’s depth.

To elucidate the behavioural parameters of murine water aversion and facilitate the development of new behavioural tests that could aid the identification of relevant neural circuits, we investigated the behaviours of mice when placed in proximity to water. We examined whether and under what conditions mice placed in a box with an area of shallow water would approach and enter the water.

## 2 Results

### 2.1 Tests with an object in the water

**Figure 1:**
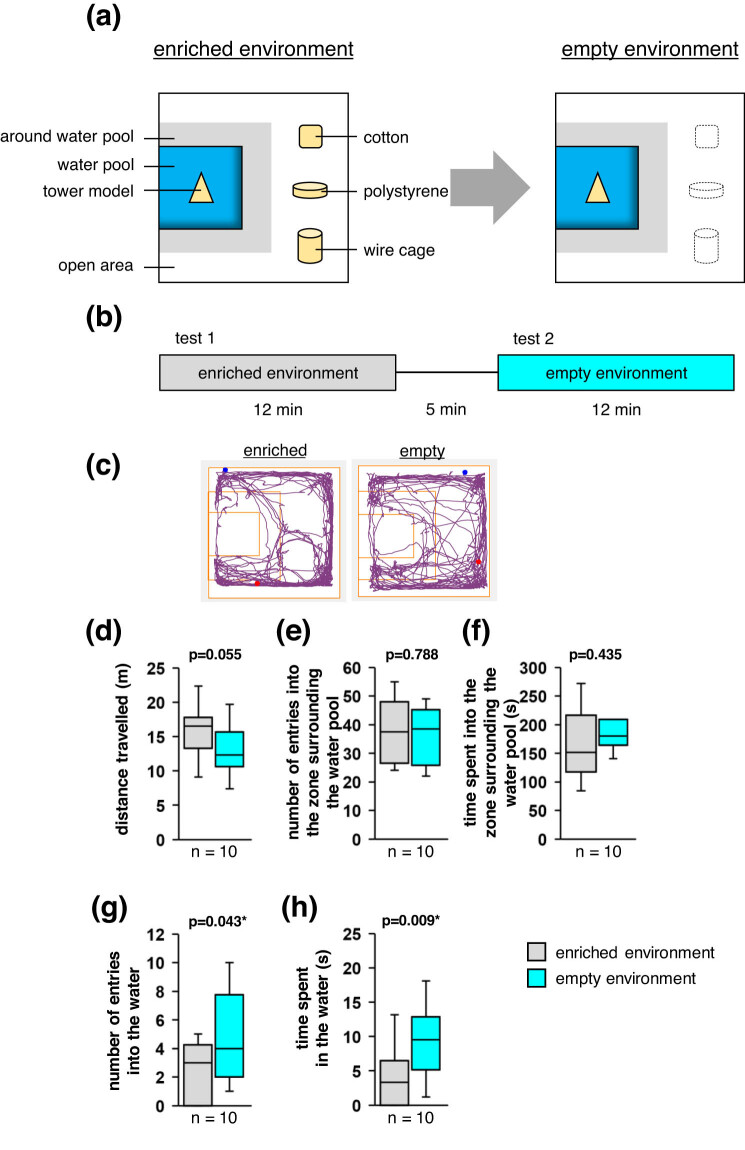
Behavioural tests featuring an object in the water pool. (a) Schematics of the experimental environments. (b) Outline of the experimental protocol. (c) Sample traces of a mouse’s movements through the enriched and empty environments. (d–h) Boxplots showing the total distance travelled (d), the number of entries into the zone surrounding the water pool (e), the total time spent in that zone (f), the number of entries into the water (g), and the total time spent in the water (h) under each experimental condition. Statistical significance was defined as *p < 0.05.

To determine whether a mouse’s interest in exploring objects could tempt it to explore a water pool containing an object, we compared the behaviours of mice in an enriched environment (i.e. one with objects present both within and outside the water pool) with their behaviours in an empty environment (i.e. one with objects only present within the water pool) (Figures 1a and b). We observed no significant between-condition differences in the total distance travelled (Figures 1c and d), the number of entries into the zone surrounding the water pool (Figure 1e), or the total time spent in that zone (Figure 1f). However, we observed that the number of entries into the water pool and the total time spent in the water were greater under the empty environment condition than under the enriched environment condition (Figures 1g and h; Supplementary Videos 1 and 2), which suggests that the mice were more motivated to explore the water pool when the water pool was the only area that contained an object.

### 2.2 Murine interest in the water pool

**Figure 2:**
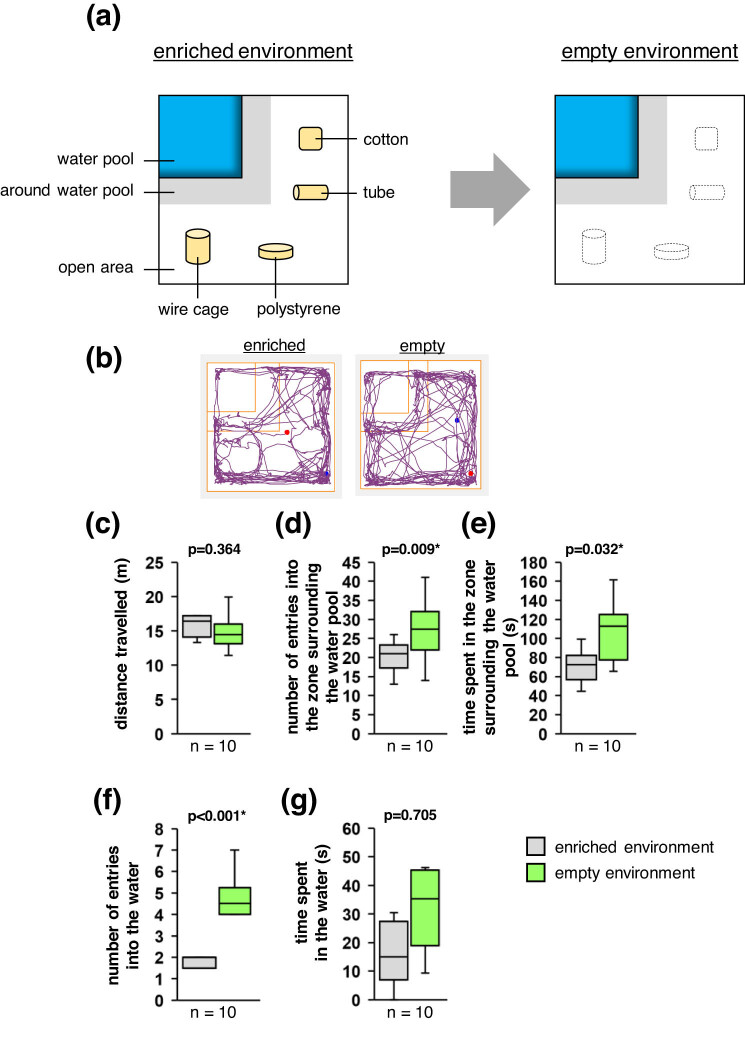
Behavioural tests without an object in the water pool. (a) Schematics of the experimental environments. (b) Sample traces of a mouse’s movements through the enriched and empty environments. (c–g) Boxplots showing the total distance travelled (c), the number of entries into the zone surrounding the water pool (d), the total time spent in that zone (e), the number of entries into the water (f), and the total time spent in the water (g) under each experimental condition. Statistical significance was defined as *p < 0.05.

To determine whether mice would exhibit any interest in exploring a water pool by itself, we compared the behaviours of mice in an enriched environment (i.e. one with objects present outside the water pool) with their behaviours in an empty environment (i.e. one without any objects) (Figure 2a). We observed no significant between-condition difference in the total distance travelled (Figures 2b and c), but we observed that the number of entries into the zone surrounding the water pool and the total time spent in that zone were greater under the empty environment condition than under the enriched environment condition (Figures 2d and e). We also observed more entries into the water pool under the empty environment condition than under the enriched environment condition (Figure 2f; Supplementary Videos 3 and 4), but we observed no significant difference in the total time spent in the water (Figure 2g). These results indicate that the willingness of a mouse to enter the water is not dependent on the presence of objects in the water pool. However, the fact that the mice spent more time walking around the water pool than in it suggests that the mice were still hesitant to enter the water.

### 2.3 Effects of variable water depths on mouse behaviours

**Figure 3:**
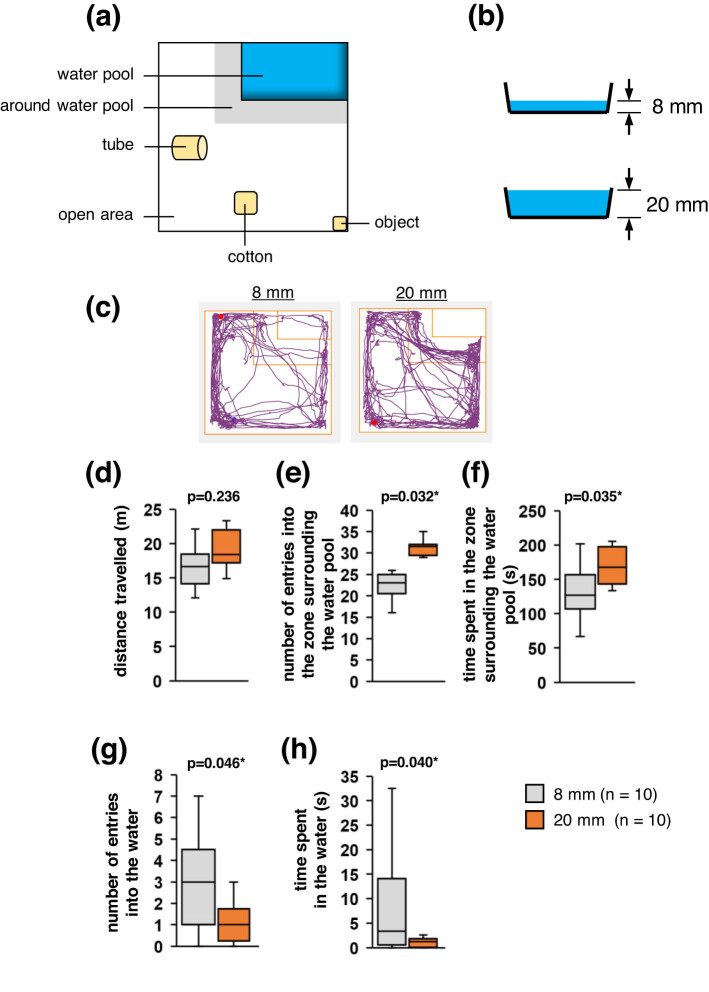
Behavioural tests with variable water depths. (a) Schematic of the experimental environments. (b) Schematic of the difference in water depths. (c) Sample traces of a mouse’s movements under the 8 and 20 mm depth conditions. (d–h) Boxplots showing the total distance travelled (d), the number of entries into the zone surrounding the water pool (e), the total time spent in that zone (f), the number of entries into the water (g), and the total time spent in the water (h) under each experimental condition. Statistical significance was defined as *p < 0.05.

To determine the effects of water depths on a mouse’s willingness to enter the water, we compared the behaviours of mice in the presence of an 8 mm-deep water pool with those in the presence of a 20 mm-deep water pool (Figures 3a and b). We observed no significant between-condition difference in the total distance travelled (Figures 3c and d), but we observed that the number of entries into the zone surrounding the water pool and the time spent within that zone were significantly greater under the 20 mm depth condition than under the 8 mm depth condition (Figures 3e and f). The number of entries into the water pool and the total time spent in the water were both lower under the 20 mm depth condition than under the 8 mm depth condition, with most mice not entering the water at all under the 20 mm depth condition (Figures 3g and h; Supplementary Video 5). These results suggest that mice can determine the depth of a pool of water.

### 2.4 Behaviours of mice surrounded by water

**Figure 4:**
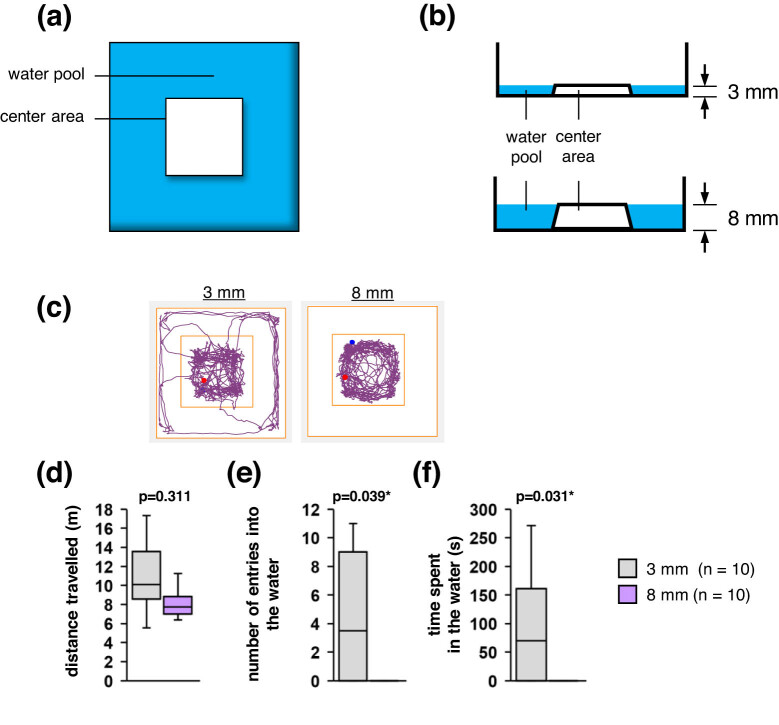
Behavioural tests with the mice surrounded by water. (a) Schematic of the experimental environments. (b) Schematic of the difference in water depths. (c) Sample traces of a mouse’s movements under the 3 mm and 8 mm depth conditions. (d–f) Boxplots showing the total distance travelled (d), the number of entries into the water (e), and the total time spent in the water (f) under each experimental condition. Statistical significance was defined as *p < 0.05.

To determine how a stressful situation affects a mouse’s willingness to enter the water, we compared the behaviours of mice surrounded by 3 mm-deep water with those of mice surrounded by 8 mm-deep water (Figures 4a and b). We observed no significant between-condition difference in the total distance travelled (Figure 4d), but we observed that the mice only entered the water under the 3 mm depth condition (Figures 4c, e and f; Supplementary Video 6). These results constitute further evidence that mice can determine the depth of a pool of water. They also indicate that being surrounded by water reduces a mouse’s willingness to enter the water.

## 3 Discussion

In this study, we have shown that a strain of mice widely used in experiments can recognise water depths, they are unwilling to enter unacceptably deep bodies of water, and the distressing situation of being surrounded by water reduces their depth tolerance.

Although the mice frequently exhibited some apparent hesitancy about entering the water, with protracted periods in which they restricted themselves to exploring the zones surrounding the water, they usually exhibited an eventual willingness to enter and explore the water pools regardless of whether objects were present within the open field device. These behaviours differ markedly from those observed in situations where mice are presumably simply attempting to avoid potential predators, such as the tendency of mice in a boxed environment to focus on exploration near the wall while avoiding the less protected open areas [16,17]. Indeed, the water-entry behaviours that we observed are more reminiscent of the risk-accepting behaviours after initial periods of risk-avoidance observed in various behavioural tests [18]. For example, in the open field test, light/dark transition test, and elevated plus maze test, mice initially avoid open areas, highly illuminated areas, and heights, but they also exhibit an eventual willingness to explore new and potentially risky spaces [19–21]. The behaviours of mice when confronted with novelty are thus determined by a conflict between the willingness to explore unknown areas and objects and the motivation to avoid potential danger. The willingness of mice in our experiments to approach and enter bodies of water probably reflects an innate motivation to explore new environments and is thus analogous to the willingness of mice to enter the open arms in the elevated plus maze test, the central area in the open field test, and the illuminated area in the light/dark transition test.

Avoidance behaviours depend on an animal’s senses and are influenced by its motor activity, motivational factors, and search strategies [22]. The elevated plus maze test, light/dark transition test, and open field test are all designed to evaluate anxiety-like behaviours by taking advantage of known avoidance behaviours [23], but the results obtained from the different tests are sometimes inconsistent [20,24–26]. Discrepancies may arise from the fact that these tests are based on distinct anxiety-like behaviours [23]: avoidance of illuminated spaces in the light/dark transition test, avoidance of open spaces in the open field test, and avoidance of heights in the elevated plus maze test [27]. The observed discrepancies suggest that different forms of anxiety may involve distinct mechanisms, and this, in turn, implies that it is important for researchers to have access to diverse tests with which to assess different forms of anxiety [28,29]. Our findings concerning the characteristics of murine water avoidance may be used to develop a new test of anxiety-like behaviours based on water avoidance.

In this study, mice exhibited a markedly reduced willingness to enter the water when the depth was increased to 20 mm, even though they could still walk through the water without needing to swim at that depth. Mice can swim but they will normally avoid water as much as possible [1]. Our results clearly show that mice can recognise the depth of a body of water and can choose to avoid entering deep water, just as experiments with elevated plus mazes have shown that mice can recognise heights and can choose to avoid them [30]. To the best of our knowledge, our study is the first to provide empirical evidence that mice are more willing to enter shallow water than to enter deep water.

Interestingly, we found that when mice were placed in a central area surrounded by water, they exhibited an increased aversion to deep water, with no entries into the water with an 8 mm depth that had been acceptable for mice that were not surrounded by water. This suggests that being surrounded by water prompted increased feelings of anxiety in the mice and reduced their willingness to engage in exploratory behaviours, a finding that is consistent with past investigations showing that anxiety suppresses exploratory behaviours [31]. Other factors, such as situation complexity, novelty, and the animal’s baseline emotional state, can also reduce a mouse’s willingness to explore a new environment [32].

Anxiety and fear are normal emotions that are selected for in the evolutionary process because they aid an organism in avoiding dangerous situations. For example, humans can experience fear around water because of the risk of drowning. However, individuals with aquaphobia experience abnormal symptoms around water such as headaches, feelings of suffocation, panic attacks, and decreased water intake [10]. Fear occurs in response to threats, but the physiological mechanisms underlying anxious behaviours remain unclear [33]. For small rodents, entering small spaces, holes, or tunnels is an important behaviour, but mice that lack leucine-rich repeat transmembrane neuronal proteins exhibit claustrophobia-like phenotypes that involve avoidance of small enclosures [34]. Interestingly, mice with hippocampal lesions also exhibit an unwillingness to enter small holes and tunnels [35]. Investigations into social phobias have found that such phobias may be related to interactions between the noradrenalinergic and serotonergic systems and the hypothalamic–pituitary–adrenal system [15,36]. Collectively, these findings indicate the existence of neural mechanisms underlying innate fears and offer clues as to how therapeutic strategies for phobias and anxiety could be developed. Our results add to the existing knowledge concerning phobias and may aid efforts to elucidate the mechanisms underlying water avoidance in mice and aquaphobia in humans.

## 4 Conclusion

Our results clearly indicate that mice exhibit exploratory behaviours in the context of entering shallow water. Furthermore, mice can recognise water depths and can choose not to enter the water if it is too deep. We speculate that the extent of a mouse’s exploratory behaviour in the presence of bodies of water is partially determined by anxieties related to water, and we propose that the dependence of a mouse’s exploratory behaviours on water depths could be used to design new tests of anxiety-like behaviours that could aid research into aquaphobia in humans.

## 5 Materials and methods

### 5.1 Animals

All efforts were made to minimise the number of animals used and to prevent unavoidable discomfort. Male C57BL/6N mice (age: 10 weeks) were purchased from Charles River Laboratories Japan (Kanagawa, Japan) and were housed five to a cage with food and water provided *ad libitum* under a 12 h light/dark cycle at 23–26°C.


**Ethical approval:** The research related to animals’ use has complied with all the relevant national regulations and institutional policies for the care and use of animals. All animal experiments were performed in accordance with the U.S. National Institutes of Health (NIH) – Guide for the Care and Use of Laboratory Animals (NIH Publication No. 80-23, revised in 1996) and approved by the Committee for Animal Experiments at the Kawasaki Medical School Advanced Research Center.

### 5.2 Behavioural tests

All behavioural tests were conducted in behavioural testing rooms between 09:00 h and 16:00 h during the light phase of the light/dark cycle. After the tests, the equipment was cleaned with 70% ethanol and super hypochlorous water to eliminate olfactory cues. Hypochlorous acid is an effective odour removal agent with a weak intrinsic odour [30,37]. The behavioural testing rooms were illuminated at a 100 lux intensity.

For the behavioural tests, we used an open field test apparatus that consisted of a 45 cm × 45 cm square area surrounded by 40 cm-high walls. The tests involved various arrangements of water pools and objects. Prior to object placement, each mouse was placed in the box for a 10 min free exploration period to produce habituation to the environment, after which the mouse was briefly returned to its home cage while object placement occurred. Unless otherwise noted, the water pools were filled during the habituation period. During the behavioural tests, data were video-recorded.

### 5.3 Tests with an object in the water pool

In this experiment, the open field included a 13.0 cm × 13.0 cm pool of 3 mm-deep water that was positioned at the centre of one wall (Figure 1a). A tower model was placed in the centre of the water pool, and a 4.0 cm × 4.0 cm cotton square, a 4.0 cm × 4.0 cm × 1.0 cm polystyrene rectangular prism, and a 4.0 cm × 8.0 cm × 4.0 cm wire cage were placed on the side of the open field opposite the water pool.

Ten mice were used in this experiment. In test 1, all objects were placed in the box (Figure 1a; enriched environment), and the mouse was placed in a corner before being allowed to move freely around the box for 12 min (Figure 1b). The mouse was then returned to its home cage for 5 min. In test 2, all objects except the tower model in the water pool were removed (Figure 1a; empty environment), and the mouse was again placed in a corner before being allowed to move freely around the box for 12 min (Figure 1b). The same mice were used in test 1 and test 2.

### 5.4 Tests of murine interest in the water

In this experiment, the open field included a 13.0 cm × 13.0 cm pool of 3 mm-deep water that was positioned in a corner (Figure 2a). A 4.0 cm × 4.0 cm cotton square, a 4.0 cm × 4.0 cm × 1.0 cm polystyrene rectangular prism, a 4.0 cm × 7.0 cm × 4.0 cm wire cage, and a 50 mL tube without a lid were placed in the areas away from the water pool.

Ten mice were used in this experiment. In test 1, all objects were placed in the box (Figure 2a; enriched environment), and the mouse was placed in a corner before being allowed to move freely around the box for 12 min. The mouse was then returned to its home cage for 5 min. In test 2, all objects were removed (Figure 2a; empty environment), and the mouse was again placed in a corner before being allowed to move freely around the box for 12 min. The same mice were used in test 1 and test 2.

### 5.5 Tests with variable water depths

In this experiment, the open field included a 14.0 cm × 20.0 cm water pool with a depth of 8 mm or 20 mm positioned in a corner (Figures 3a and b). The same container was used for all experiments to ensure equal container heights. A 4.0 cm × 4.0 cm cotton square, a 50 mL tube without a lid, and a third object (miniature-home) were placed in areas away from the water pool. Separate sets of 10 mice were used for tests involving the 8 mm and 20 mm depths. In this test, the mouse was placed in the corner before being allowed to move freely around the box for 12 min. In this experiment, each mouse was used in only one experiment.

### 5.6 Tests with water surrounding a dry zone

In this experiment, the open field consisted of a 13.0 cm × 13.0 cm dry central area that was surrounded by a water pool with a depth of 3 mm or 8 mm (Figures 4a and b). Separate sets of 10 mice were used for tests involving the 3 mm and 8 mm depths. In contrast to the other experiments, water was not added to the box until after the 10 min habituation period. In this test, the mouse was placed on the dry central area before being allowed to move freely around the box for 12 min. In this experiment, each mouse was used in only one experiment.

### 5.7 Data analyses

The video-recorded data were analysed with video-tracking software (ANY-MAZE; Stoelting, Wood Dale, IL, USA). For each 12 min test period, we determined the total distance travelled, the number of entries into the zone surrounding the water pool, the amount of time spent in that zone, the number of entries into the water pool, and the amount of time spent in the water pool. For comparing two groups, Student’s *t*-test was used for normally distributed data, and Mann–Whitney *U* test was used for not normally distributed data. In addition, one-way repeated-measures analysis of variance was used for normally distributed data, and the Friedman test was used for not normally distributed data. *p* < 0.05 was used as the definition of statistical significance. Statistical analyses were performed with SPSS software (IBM, Armonk, NY, USA).
